# Efficiency of Mobile Video Sharing Application (WhatsApp®) in Live Field Image Transmission for Telepathology

**DOI:** 10.1007/s10916-020-01567-w

**Published:** 2020-05-02

**Authors:** Rituparna Das, Nidhi Manaktala, Tanupriya Bhatia, Shubham Agarwal, Srikant Natarajan, Amitha Juanita Lewis, Shweta Yellapurkar

**Affiliations:** 1 Manipal College of Dental Sciences, Manipal Academy of Higher Education, Mangalore, India; 2 Department of Oral Pathology and Microbiology, Manipal College of Dental Sciences, Manipal Academy of Higher Education, Mangalore, India

**Keywords:** Telepathology, Medical information applications

## Abstract

Telepathology is in its nascent stages in India. Video calling applications in mobile phones can be efficiently used to transmit static and live field microscopic images hastening low cost telepathology. To evaluate the efficiency of WhatsApp® Video Calling for dynamic microscopy in distant diagnosis. Thirty haematoxylin and eosin stained slides of common pathologies were retrieved from the archives of Department of Oral Pathology and Microbiology, coded with relevant history and given to three untrained investigators. The investigators then connected a mobile phone with VOIP facility to a microscope using a custom adaptor. Dynamic fields were transferred to three independent pathologists via WhatsApp® video call. The pathologists attempted to diagnose the lesion based on the live field video over their display screen (phone). Audio quality was found to be better than that of video. In 70% of the cases, pathologists could render a diagnosis (13% gave a confirmed diagnosis, 57.7% gave a probable diagnosis). The average time taken for connecting the adaptor, connecting the call to the pathologist and then receiving the diagnosis was 9:30 min. In addition, proper history taking and staining of the tissue slides were critical to arrive at the diagnosis. WhatsApp® free VOIP facility helped untrained investigators to send the live-field pathologic fields to a specialist rendering histopathological diagnosis. The factors affecting the diagnosis included network stability, clarity of images transmitted, staining quality and contrast of nuclear details of the stain. The history, clinico-pathologic correlation, transmission of static images, training of the person transmitting the images plays a vital role in rendering accurate diagnosis. Telepathology over WhatsApp® video calling could be used as an efficient screening tool to identify suspicious lesions and follow-up critical cases.

## Introduction

Telepathology is a mode of digital pathology which involves exchange of static images of photomicrographs for the purpose of histopathological diagnosis. It is a practice whereby pathologists render diagnosis remotely by viewing electronically transmitted images rather than by examination of glass slides by themselves using a light microscope. In a survey of 247 histopathologists regarding telepathology, Chordia et al. (2016) observed that digital pathology was popular and preferred in 34% of the pathologists’ practice. They further added that this was mainly for teaching purpose. The same cohort felt that in 82% of the cases telepathology would be helpful in second opinion while 67% of them felt that it would be cost effective [1]. Telepathology is often confused with the term “virtual microscopy”. Dynamic telepathology, in true sense refers to remote robotic operation of a motorized microscope and real time transmission of the video image. Virtual microscopy on the other hand involves digitization of the entire slide in high magnification which is stored in a server and can be utilized for diagnosis, teaching etc. [2, 3]

The use of robotic microscopes and image transmission systems require dedicated laboratories with high bandwidth internet connection. These high-end systems are costly. Telepathology over free to use voice and video calling applications like Skype® and MSN® have been tried out in past. These can have the advantage of being cost effective, and easy to implement [4, 5].

WhatsApp® Messenger is a freeware and cross-platform instant messaging service for smartphones. It uses Internet to make voice calls as well as one to one video calls along with exchange of text messages, images, videos, documents, user location, audio files, phone contacts and voice notes using standard cellular mobile numbers [6].

While smartphones have become a part of every individual and WhatsApp application is an integral application used by the masses owing to the versatility in sharing documents, images and it has the added benefit of being free! The aim of the present study was to harness the video calling facility provided by this application for dynamic microscopy and use it to check the efficiency in distant diagnosis.

## Materials and methods

Thirty haematoxylin and eosin stained slides were retrieved from the archives of Department of Oral Pathology and Microbiology, MCODS, Mangalore by a trained pathologist (SY). The slides were then coded with a relevant history and given to three investigators (RD, TB, and SA), making them as well as three independent pathologists (SN, NM, AL) blind to the diagnosis. The thirty slides retrieved consisted of common pathologies of epithelium, connective tissue, odontogenic origin (cysts and tumors), bone, salivary glands and skin. A Moto G4 Plus smart phone with a rear camera featuring resolution of 16 Megapixels was used. It was connected to an Olympus CH20i microscope using a custom designed adaptor. The coded slides were then focused by the investigators who then contacted the pathologist located in the adjacent room (without direct visual/audio contact with the investigators) via WhatsApp video call. The pathologists used Samsung J7 mobile phone with a display size of 5.5 in. and a resolution of 720 × 1280 pixels. During this WhatsApp video call the investigators adjusted the field of view/region of interest as per the instructions given by the pathologist via the call connected. To standardize the procedure and remove the bias of network related transmission issues the image transmission phones were connected to the same Wi-Fi router allowing a data transfer of 4 Mbps. For each case the procedure was evaluated by the investigators as per Table 1. Similarly, the quality of images, video, voice and the ability to diagnose the cases were evaluated by the pathologists as per Table 2. The analysis of the results was done using tests of proportion and frequency distribution. Following the procedure, the pathologists were interviewed regarding feedback. Problem associated with the diagnosis were discussed and documented (Tables 3 and 4).

**Table 1:**
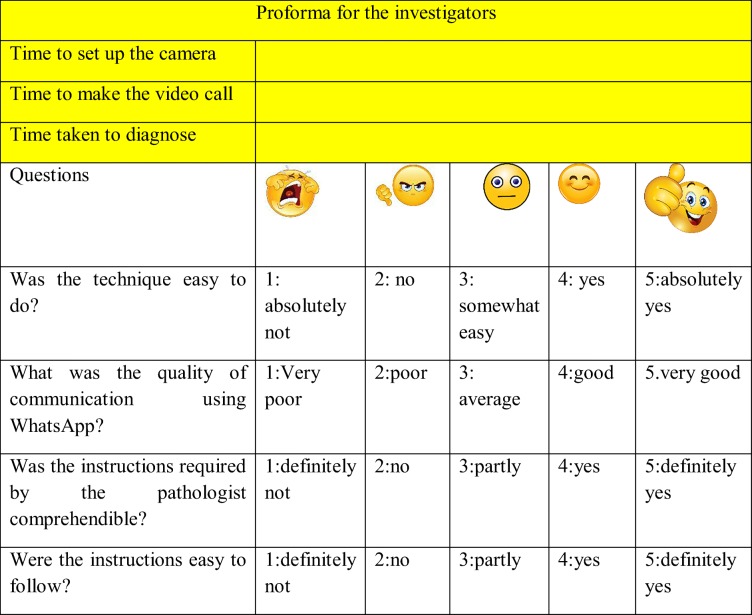
Evaluation Proforma of investigators

**Table 2:**
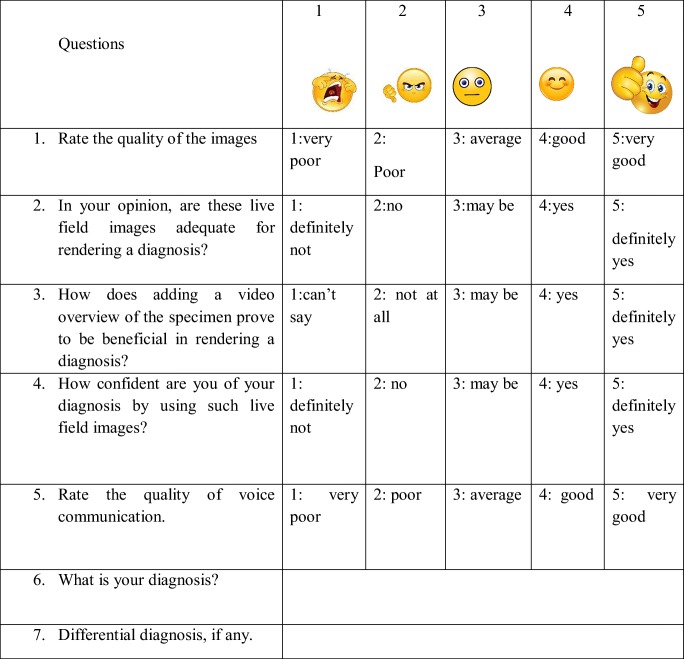
Evaluation Proforma of pathologists

**Table 3:** Assessment of the pathologists assessing the slide fields

		Frequency	Percentage
Rate the quality of the video images	Very poor	1	3.3%
	Poor	0	0.0%
	Average	16	53.3%
	Good	13	43.3%
	Very good	0	0.0%
Rate the quality of voice communication	Very poor	0	0.0%
	Poor	0	0.0%
	Average	4	13.3%
	Good	26	86.7%
	Very good	0	0.0%
Are these adequate for rendering the diagnosis	Definitely not	1	3.3%
	No	8	26.7%
	May be	17	56.7%
	Yes	4	13.3%
	Definitely yes	0	0.0%
How does adding video prove to be benificial	Definitely not	0	0.0%
	No	1	3.3%
	May be	16	53.3%
	Yes	13	43.3%
	Definitely yes	0	0.0%
How confident are you of the diagnosis	Definitely not	2	6.7%
	No	8	26.7%
	May be	15	50.0%
	Yes	5	16.7%
	Definitely yes	0	0.0%

**Table 4:** Assessment of the investigators transmitting the images

		Frequency	Percentage
Easy of technique	Absolutely not	0	0.0%
	No	0	0.0%
	Somewhat easy	0	0.0%
	Yes	27	90.0%
	Absolutely yes	3	10.0%
Quality of communication	Very poor	0	0.0%
	Poor	1	3.3%
	Average	4	13.3%
	Good	9	30.0%
	Very good	16	53.3%
Was the instruction comprehendible?	Defintely not	0	0.0%
	No	0	0.0%
	Partly	4	13.3%
	Yes	4	13.3%
	Defintely yes	22	73.3%
Instruction of pathologist easy to follow?	Defintely not	0	0.0%
	No	0	0.0%
	Partly	10	33.3%
	Yes	18	60.0%
	Defintely yes	2	6.7%

## Results

**Fig. 1:**
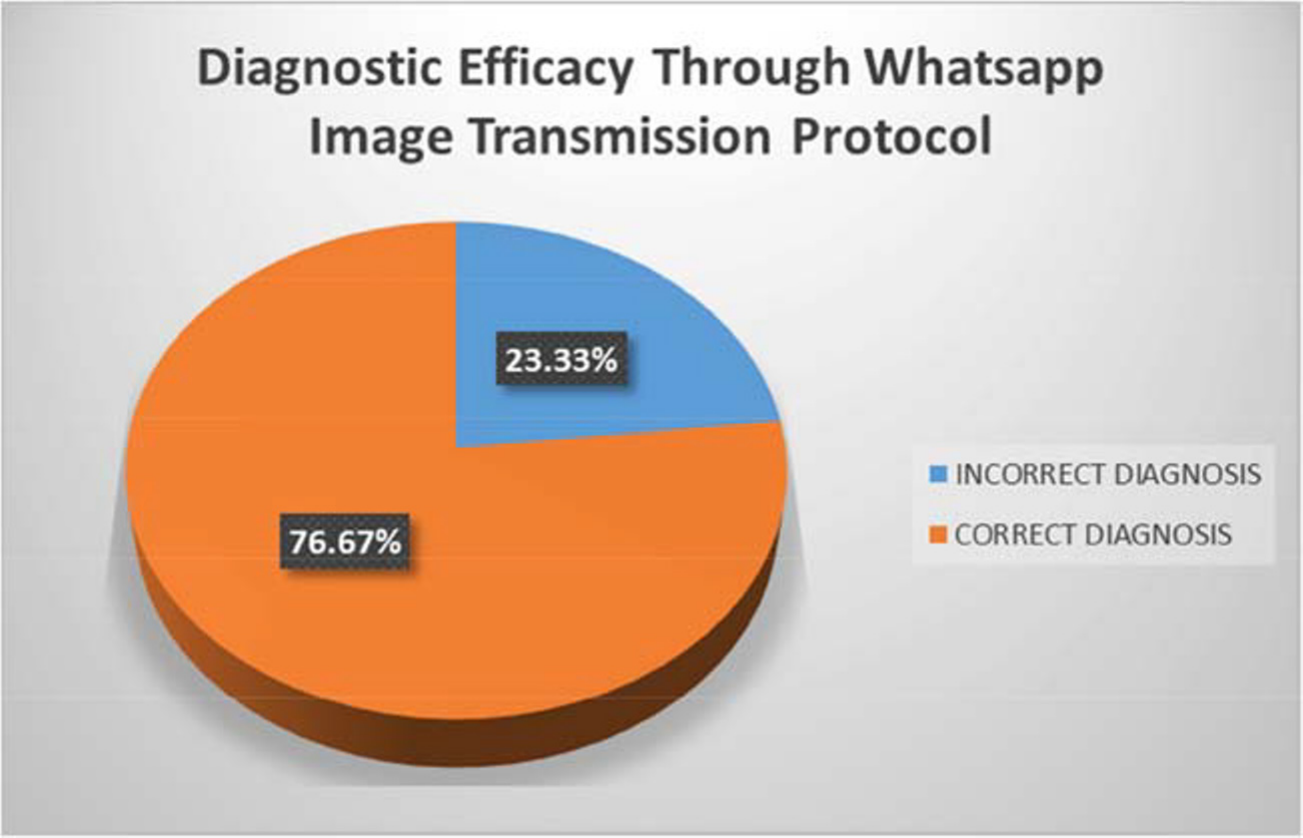
Diagnostic efficacy through WhatsApp transmission image protocol

The pathologists on their examination of the live field WhatsApp® images of the slides rated the image quality to be predominantly average to good with a percentage of 53.3% and 43.3% respectively. Additionally, they felt that the real time field assessment is adequate for rendering the assessment in only 13.3% cases. In 56.7% cases they were able to render diagnosis, but required confirmation, and in 26.7% cases it was not possible to render the diagnosis. Compared to the static field images which were generally sent for opinions, the pathologists reported that addition of a live streaming video might improve diagnosis in 53.3% cases; 43.3% of the times definitely the dynamic field was better for diagnosis. The pathologists were confident of the diagnosis in 16.7% of the cases, and in 50.0% of the cases they felt may be they were right. In the remaining 33.4% cases, they were not confident of the diagnosis. Compared to the video images the voice clarity was better rated with good or average given by the pathologists in 86.7% and 13.3% cases respectively. Final assessment of the diagnosis showed that the pathologists were able to render the correct diagnosis in 76.7% of the cases (Fig. 1).

**Fig. 2:**
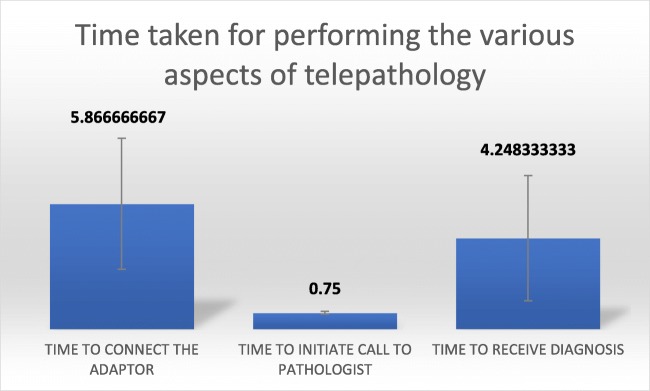
Time taken for performing the steps of telepathology (in minutes)

Evaluation of the experience of the investigators transmitting the live field, revealed that in terms of ease of technique they found the technique to be easy in all the cases. (90.0% being yes and 10.0% being an absolute yes). After the transmission of the live field WhatsApp images of the slides, the investigators rated the quality of communication to be predominantly very good to good with a percentage of 53.3% and 30.0% respectively. They were able to comprehend the instructions of the pathologist to handle and transmit the region of interest in most of the cases definitely in 73.3% of the cases. To translate the instructions into action in terms of field acquisition and focusing was possible in 66.6% of the cases (60% yes and 6.7% yes). However, in 33.3% of the cases the instructions were only partly understood and reciprocated. The average time taken for connecting the adaptor and initiating the call to the pathologist was 5.87 ± 3.07 min and 45 ± 3 s respectively. The average time taken to receive the diagnosis was 4.25 ± 2.94 min with a median of 3.81 min (Fig. 2).

## Discussion

WhatsApp® is a freeware and cross platform instant messaging and Voice Over Internet Protocol (VOIP) service founded by Brian Aeton and Jan Koun (former employees of Yahoo) in the year 2009. Initially the application allowed text, image-based message sharing between two contacts, but later the application widened its resources by providing Voice calls, Video calls, Documents and User location sharing. The application runs from a mobile device though also allows access through a computer by a web-based interface. The video calling feature was introduced across all platforms of Android, iPhone and Windows in the year 2016, by which time it was already popular among millions of users [6].

In the clinical and health care segment the adjunct diagnostic features include, clinical features, radiographs, CBCT, CT, MRI images etc. These features are essential for clinicopathologic correlation for a histopathological diagnosis of biopsy specimen. The practice of sharing these images to the pathologist/clinician are in vogue among the health care providers. Sharing of these images facilitate discussion of cases and obtain second opinion.

Telemedicine is the remote delivery of healthcare services, Such as health assessments or consultations, over the telecommunications infrastructure. It allows healthcare providers to evaluate, diagnose and treat patients without the need for an in-person visit. In line with this, telepathology is the practice of pathology at a distance. It uses telecommunications technology to facilitate the transfer of rich image pathology data between distant locations for the purposes of diagnosis, education, and research. Virtual microscopy is a technique where in a whole microscopic slide is converted to a digital file and transmitted for diagnosis. This system is costly as we need a robotic microscope along with high storage space and high bandwidth internet for transmission [3].

Dynamic telepathology through various free softwares’ allowing video over internet applications can save the cost of the telepathology. Klock C and Gomes R de PX (2008) evaluated the capture system of MSN and Skype. They used a microscope Nikon E400, trinocular and double head YTHF with a digital Samsung Color SCC-131n Camera to capture the images. The camera image was transmitted to a personal computer Quad Core Intel 3.4GHz Xeon processor, Hard disk SATA of 1 Tb, 7.200 RPM, and Windows XP SP2 as the operating system. After the establishment of connection between two pathologists a live image was seen by the receptor pathologist who was able to ask for moving the field or increase/decrease the augmentation. Both programs allowed voice transmission concomitant to image, so the communication between the involved pathologists was possible using microphones and speakers. Hence, they proved the viability of these systems to be used in developing countries and in places where there are no pathologists [4].

In the present study, we explored the possibility of dynamic video sharing for diagnosis using WhatsApp® application. We used a custom designed adaptor for android smart phones to connect to a microscope and utilized this to transmit dynamic field of microscopic slide via the video call to the pathologists. This has been done by other researchers previously. Ekong D et al. (2017) used a 3-D printed smartphone adapter for iPhone 5 model and acquired static images and videos of the slides and the images where submitted to the pathologists. Each reviewer evaluated certain questions related to quality of the images, adequacy for rendering a diagnosis, confidence in establishing the diagnosis or differential diagnosis. Most of the participants found that 8 MP camera was sufficient for evaluation although the 16 MP camera was better for video [7]. .Sarode et al. suggested the use of a printed grid template to be placed under the slide to guide the reporting pathologist. This would help the pathologist to guide the sender to click and send pictures of areas of interest in a histo-pathologic sections [8]. In our study, a 16 MP camera was transmitting the images and was received in phone capable of rendering high definition quality of images in an AMOLED screen.

Innovations in pathology practice have led to the creation of an app “Pocket Pathologist”, wherein WhatsApp® has been used to facilitate rapid diagnostic pathology teleconsultation utilizing a smartphone. The iPhone App affords an easy solution for global users to submit digital pathology images to pathology experts for consultation [9]. Sahin et al. demonstrated the efficacy of Whatsapp® in telecytology whereby easy, fast, and high-quality image capturing and transfer was possible from cytology slides using smartphones [10]. Goyal et al. (2017) have also reported the efficiency of the WhatsApp® messenger in teaching postgraduate pathology students. Their study involved discussion of 16 pathology cases with 647 posts and was well received by the postgraduates. This study highlighted the efficiency of such web tools in improving pathology teaching [11].

Giordano V et al. (2017) reviewed the literature pertaining to WhatsApp® in Telemedicine. They found a total of 30 studies of which 5 were relevant in the medical field. Their review showed that WhatsApp® is a promising means of communication between health care professionals, radiologists, and general public both in-terms of learning and information. They reported that issues of ethics and security were not discussed in any of the studies they reviewed, and that new users of WhatsApp must consider cyber security and the ethical implications of telehealth [12]. In the present study, WhatsApp® video calling feature was used for diagnosis of pathology slides. To the best of our knowledge this is the first study which utilizes live feed video over internet for histo-pathological diagnosis. Fontelo et al. (2015) reported that the use of a video overview in addition to the photographs, improved diagnostic confidence [13]. In our experience WhatsApp® based communication had better clarity in audio than video transmission. The investigators felt in 83.3% of the cases that the audio quality was good to very good and the audio was clear and comprehendible. In 33% of the cases the investigators found it partly difficult to follow the pathologist’s instructions. It is thus, recommended to use earphones for such a communication as the clarity and focus (reduced interference of background noises) was better improving comprehension and following of instructions.

The manner in which the connecting phone and receiving phone were oriented with the microscopic field and with each other played a critical role in communication. The direction of left-right-up-down were different for the pathologist as the two communicating phones were oriented perpendicular to each other. Thus, the pathologist needs to understand the direction of movement of slide for correct instructions to the person transmitting the images.

During the experiment, the pathologists felt it easier to designate the tissue type or pattern and give instructions to the person transmitting the images to focus the field of interest. It is thus emphasized that if the person transmitting the images has basic knowledge of the various tissue types, nuclear and cellular features it would be easier to communicate and follow the instructions. It is also imperative to have a good wireless connection with high speed internet to enable high resolution videos to be transmitted. Fifty three percent of the pathologists considered the quality of images to be of average quality. Even though both the investigators transmitting the image and the pathologists receiving the image were connected to the same Wi-Fi router the quality was not found to be adequate for diagnosis in 26.7% of the cases. Our findings are in sync with the study by Alfaro L. and Roca MJ (2008), who demonstrated that portable telepathology was a cost-effective method of diagnosis but depends on the standardized network bandwidth to achieve maximum image quality [5]. However, image quality can be enhanced using a high-resolution image sharing (HIS) technique, wherein photomicrographs can be sent as documents in contrast to a conventional image (where the resolution is compromised). This could serve as an aid to video streaming the quality of which always depends on the bandwidth [14].

Liang et al. (2008) found that the second opinion diagnosis of cases using telepathology required lesser average time for diagnosis as compared to the interdepartmental consultation. They found good correlation between the direct and the telepathology diagnosis. In 18% of the cases they found telepathology consultation resulted in a positive major impact on the diagnosis [15]. In our study the average time taken for connecting the adaptor, connecting the call to the pathologist and then receiving the diagnosis was 9–10 min, which is reasonable for screening and first impression.

An interview with the pathologists highlighted important findings other than network, which affect diagnosis. The pathologists agreed that the history and presentation of the lesion were critical for the diagnosis; without the history the accuracy and confidence would be much lower than 76.67% and 66.7% respectively as noted in the present study. Staining of the tissue slides was equally critical in diagnosis. Numerous epithelial and connective tissue patterns were perceivable when the slides were well stained slide with a good contrast between the cytoplasm and the nucleus. The misdiagnoses on telepathology can be credited to sampling error, choosing non-representative microscopic fields, misinterpretation as a result of poor image quality, or misinterpretation not attributable to image quality [16]. Garg et al. evaluated the utility of WhatsApp image transfer in the histo-pathological diagnosis of common oral malignant and benign lesions. In their study, they included a total of 100 cases of oral biopsy which comprised of 58 cases of squamous cell carcinoma, 33 cases of oral leukoplakia, 3 cases of oral lichen planus and 6 cases of moderate to severe dysplasia. They concluded despite having an encouraging overall concordance rate of 95%, there was still a long way to go for a routine use of diagnostic telepathology in onco-pathology. They also stressed upon the pathologists need to learn where to draw the line and ask for glass slides for final diagnosis [17].

The pathologists were not able to render the correct diagnosis in conditions where nuclear features played a critical role in diagnosis. The pathologists were not able to appreciate nuclear atypia in cases of dysplasia and squamous cell carcinoma. Some of the slides examined had poor staining which led to lack of confidence in identifying a particular tissue type for example, a poorly stained epithelial tissue mass resembled a necrotic or a bone tissue. Addition of a blue filter in certain cases improved the contrast and thereby rendition of the diagnosis.

## Conclusion

WhatsApp® video communication with a custom-built adaptor is a viable alternative for low cost telepathology. The video and the audio communication allowed examination of the photomicrographs and dynamic live stream video enabling the pathologists to arrive at a diagnosis. Although possible better network would improve the quality of images. The diagnosis could be improved by addition of the relevant clinical history, static adjunct images and a good staining. Basic training of individuals involved in transmitting the images in terms of patterns of pathology and basic tissue types could improve the instructions and communication.

In future, setting up a laboratory with minimal equipment required for tissue processing, sectioning and staining accompanied by a low cost telepathology unit could be a viable alternative for screening and diagnosis. In high end hospitals and research units telepathology could be a tool to obtain second opinions. Simultaneously, WhatsApp® images and videos can be viewed and downloaded on a computer for integration with electronic medical records [18]. With the advent of 4G even at remote locations, the use of WhatsApp has already been tried in facilitating remote oral medicine consultation [19]. In conclusion, we completely agree with the findings of Dorwal et al. (2017), that telepathology using WhatsApp® contributed to significant improvement in the communication in the form of sharing photographic evidence and academic activities. There was also some increase in the load of adding information to the application and disturbance in the routine activities; but the benefits far outweighed the minor hassles [20].

The present study validates the fact that careful use of Whatsapp® or any other message transmission applications for that matter, can significantly aid in improving communication across the laboratory/ diagnostic services, thereby ensuring timely intervention/ action (where required), ultimately rendering improved patient care and quality of healthcare services. At the same time, attention must be given to confidentiality, consent and data security such that misuse of data doesn’t take place.
